# Characterization of the stress level of university students using data mining algorithms

**DOI:** 10.3389/frma.2025.1637206

**Published:** 2025-11-21

**Authors:** Yuri Reina Marín, Lenin Quiñones Huatangari, Omer Cruz Caro, Einstein Sánchez Bardales, Judith Nathaly Alva Tuesta, Jorge Luis Maicelo Guevara, River Chávez Santos

**Affiliations:** 1 Office de Gestion de la Calidad, Universidad Nacional Toribio Rodriguez de Mendoza de Amazonas, Chachapoyas, Peru; 2 Facultad de Ingeniería Zootecnista, Biotecnología, Agronegocios y Ciencia de Datos, Universidad Nacional Toribio Rodríguez de Mendoza de Amazonas, Chachapoyas, Peru; 3 Pontificia Universidad Catolica del Peru, Lima, Peru

**Keywords:** academic stress, data mining, college students, ranking rule, stress prediction, stress coping

## Abstract

There is concern about the levels of stress faced by college students and their effects on mental health and academic performance. This study aimed to characterize academic stress levels in college students, using data mining algorithms to classify and predict risk patterns. Data were collected from 287 students using the SISCO Academic Stress Inventory, and classification algorithms and association rules were applied using WEKA software. The results revealed that 75.3% of the students experienced high stress levels, primarily linked to psychological reactions and academic demands. It also compared the predictive performance of 13 algorithms, where J48, LMT, and SimpleLogistic achieved classification accuracies above 89%, surpassing results previously reported in similar educational contexts. Association rule mining further showed that being single and childless was strongly correlated with elevated stress levels, highlighting demographic risk profiles often overlooked in earlier research. By integrating predictive modeling with demographic and behavioral factors, this study extended prior literature by showing how data mining can simultaneously classify and explain academic stress, offering actionable insights for universities to design targeted, evidence-based interventions.

## 1 Introduction

A growing number of young people pursue higher education seeking to strengthen their academic foundation and enhance their opportunities in an increasingly competitive labor market (Carrasco et al., 2024; Sánchez et al., 2024; Xiao et al., 2024). However, this educational process unfolds within a context of high academic demands, economic uncertainty, and limited professional opportunities, which can generate high levels of stress and anxiety (Leslie et al., 2021; Noman et al., 2021). The transition to university life is also a crucial stage of personal development, involving major changes in habits, lifestyle, and support networks, along with the challenge of adapting to a new academic system (Durán Acevedo et al., 2021). If unmanaged, these conditions may trigger mental health problems that directly affect academic performance, persistence, and overall student wellbeing (Castillo-Navarrete et al., 2024; Gogichadze et al., 2023).

Higher education benefits both individuals and society as a whole (Hitches et al., 2022), nevertheless, the academic journey presents numerous challenges (Durán Acevedo et al., 2021). Internationally, students report high stress levels related to their education, which can negatively impact health, quality of life, and academic achievement (Pascoe et al., 2020; Powell and Graham, 2017). However, academically confident students experience less stress, adapt more successfully to college, and are generally considered healthier and happier individuals (Chemers et al., 2001). Success in higher education encompasses both academic achievement and life satisfaction, which can be predicted by academic confidence (self-efficacy) and stress (Krumrei-Mancuso et al., 2013). Therefore, examining the latter factors may provide information on how best to support college students to reach their full potential and which individuals may need support (Hitches et al., 2022).

University students tend to experience higher levels of stress and anxiety compared to the general population (Bewick et al., 2010; Bonneville-Roussy et al., 2017; Deasy et al., 2015). In the United Kingdom, mental health problems have increased fivefold (Thorley, 2017); in Asia, 11% of students report stress and anxiety (Cuttilan et al., 2016); and in Malaysia, 47.1% exhibit decreased psychological wellbeing (Zulkefly and Baharudin, 2010). There has been growing research interest in stress and coping strategies, particularly among medical students (Melaku and Bulcha, 2021; Tran et al., 2023; Uyen et al., 2024). Among Vietnamese medical students, more than 30% report moderate to high stress (Nguyen-Thi et al., 2024; Pham et al., 2023). Prolonged exposure to high stress leads to cognitive and emotional overload, psychological distress, school dropout, poor quality of life, and reduced empathy, with professional risks such as medical errors (Gleichgerrcht and Decety, 2013; Ruzhenkova et al., 2018; Wong and Chapman, 2023).

Research suggests that college students experience stress from multiple sources, academic, psychosocial, and financial, often related to professor, student relationships and high personal or external expectations (Ragab et al., 2021; Saxena et al., 2014). Physical problems can also be significant stressors affecting academic performance and quality of life (Coffin et al., 2023; Maity et al., 2022). Academic-related stressors in particular have been found to cause more distress than interpersonal, intrapersonal, or environmental ones (Sadiq et al., 2021). Early identification of these stressors may help design effective prevention and intervention programs aimed at reducing psychological problems among this high-risk population (Klein and McCarthy, 2022; Slavin et al., 2012).

Students tend to experience high stress, especially in their first year, due to difficulties balancing studies, work, and family responsibilities, as well as limited knowledge of the teaching profession (Geng and Midford, 2015). Exams, assessments, and professional practice requirements increase this burden (Deasy et al., 2015; Gustems-Carnicer et al., 2019), negatively affecting academic performance (Gustems-Carnicer et al., 2019). Female students often report higher stress than males (Deasy et al., 2015). while the effect tends to decrease with age (Gustems-Carnicer et al., 2019). Time management and self-regulation strategies can reduce stress and anxiety (Heikkilä et al., 2012), yet many students are reluctant to seek help or do not know how to access support (Deasy et al., 2015; Geng and Midford, 2015). This highlights the importance of timely interventions in higher education to better understand and address students' needs (Hitches et al., 2022).

There are studies on academic stress focused on the use of machine learning to predict stress levels based on academic performance and study load (Shahapur et al., 2024), artificial neural networks to estimate mental health (Pei, 2022), and classification algorithms such as Support Vector Machine (SVM), Decision Tree (DT), and Random Forest (RF) to detect mental stress by evaluating factors such as internet use, academic workload, exam pressure, and family environment (Ahuja and Banga, 2019; Firoz et al., 2023; Wicaksono and Sriani, 2024). Data mining has proven to be a tool for characterizing academic stress in students by identifying stressors and offering the possibility of early interventions (Anand et al., 2023). The application of this methodology in the field of academic stress remains scarce; therefore, the proposed study seeks to fill this gap by using data mining, applying algorithms to classify and predict stress levels, and using association rules to reveal hidden relationships between variables, which will allow a better understanding of the factors that influence academic stress.

## 2 Materials and methods

### 2.1 Place of study

The study population comprised students from the 2nd to 12th−14th academic cycles of the different professional careers of the National University Toribio Rodriguez of Mendoza of Amazonas, enrolled in the 2024-II academic semester.

### 2.2 Methodology

**Figure 1:**
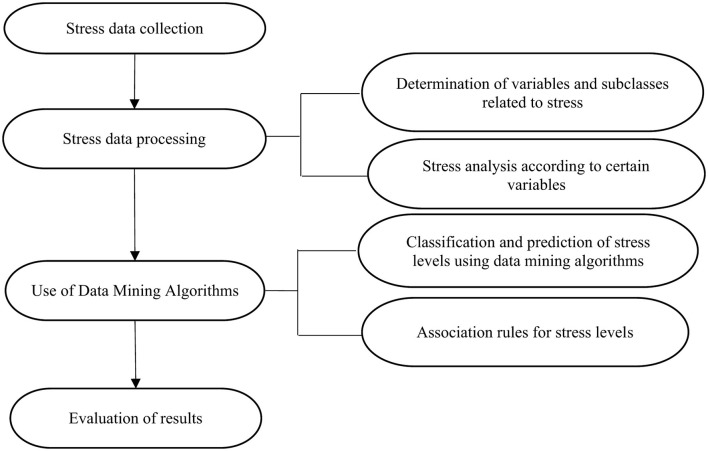
Study methodology.

The study analyzed stress levels among students enrolled at the National University Toribio Rodriguez of Mendoza of Amazonas during the 2024-II semester. Data collected on students' stress levels were analyzed using data mining algorithms implemented in the open-source software WEKA (version 3.8.6; see Figure 1). Although this cross-sectional approach offers strong descriptive and predictive value, it does not capture variations in stress levels across semesters, a limitation that should be considered when interpreting the results.

#### 2.2.1 Data collection

Data were collected through an online survey distributed via institutional emails to students and faculty in May, coordinated by the university's Office of Information Technology. The primary instrument used was the SISCO Academic Stress Inventory developed by Guzmán-Castillo et al. (2022), was applied as the main instrument. The questionnaire was structured in sections: (I. general data, II. stressors, III. physical, psychological, and behavioral reactions when you were worried or nervous, IV. stress coping strategies). This questionnaire was designed on a Likert scale where (1) is never, (2) is rarely, (3) is sometimes, (4) is almost always, and (5) is always. The questionnaire demonstrated high internal consistency, with a Cronbach's alpha coefficient of 0.89.

The data set used in the study includes students enrolled in semester 2024-II. Table 1 provides information on variables affecting academic stress during the semester under study. Detailed information on the variables was obtained using a survey, for which the participants gave informed consent. A total of 383 students participated in the survey, providing a robust dataset for subsequent analysis.

**Table 1:** Description of variables affecting academic stress.

**No**.	**Variables**	**Description**	**Type**	**Range**
1	Study area	Determine the area of study according to the professional career	Categorical	(1) Engineering (2) Social sciences (3) Accounting, economics, and administrative sciences (4) Health sciences
2	Training cycle	Identifies the student's academic cycle according to the range of university education	Categorical	(1) General training (cycles I to III) (2) Specific training (cycles IV to VII) (3) Specialty training (cycles VIII to XII–XIV)
3	Number of courses enrolled	Determine the number of courses enrolled in semester 2024 II per student	Discrete	
4	Hours of study	Determine the weekly hours spent by students studying outside of class time	Discrete	
5	Age	Identifies the student's age	Discrete	
6	Gender	Identifies students' biological gender	Categorical	(1) Female (2) Male
7	Marital status	Identifies students' marital status	Categorical	(1) Single (2) Cohabitant (3) Married
8	Number of children	Identifies the number of students' children	Discrete	
9	Academic performance	Identifies the student's weighted average for the semester 2024 I	Continuous	
10	Level of concern or nervousness	Determines the student's level of concern or nervousness	Categorical	(1) Little (2) Some (3) Moderate (4) Quite (5) Much
11	Level of stressors	Determine the level of stressors that influence student stress	Categorical	(1) Low (2) Medium (3) High
12	Physical reactions	Determines the level of stress-related physical reactions of the student	Categorical	(1) Low (2) Medium (3) High
13	Psychological reactions	Determines the level of stress-related psychological reactions of the student	Categorical	(1) Low (2) Medium (3) High
14	Behavioral reactions	Determines the level of stress-related behavioral reactions of the student	Categorical	(1) Low (2) Medium (3) High
15	Strategy 1	Ability to defend our preferences or feelings without harming others	Categorical	(1) Never (2) Rarely (3) Sometimes (4) Almost always (5) Always
16	Strategy 2	Development of a plan for the execution of your tasks	Categorical	(1) Never (2) Rarely (3) Sometimes (4) Almost always (5) Always
17	Strategy 3	Self-praise	Categorical	(1) Never (2) Rarely (3) Sometimes (4) Almost always (5) Always
18	Strategy 4	Religious practice (prayers or church/temple attendance)	Categorical	(1) Never (2) Rarely (3) Sometimes (4) Almost always (5) Always
19	Strategy 5	Search for information on the situation	Categorical	(1) Never (2) Rarely (3) Sometimes (4) Almost always (5) Always
20	Strategy 6	Ventilation and confidence (verbalization of the situation that worries)	Categorical	(1) Never (2) Rarely (3) Sometimes (4) Almost always (5) Always
21	Stress level	Determines the stress level of university students	Categorical	(1) Low (2) Medium (3) High

The class label (Stress level) was derived from the SISCO Academic Stress Inventory. Following the validated scoring procedure, total scores were computed and categorized into three levels: low, medium, and high. These categories are based on the standardized cutoffs of the instrument, not on students' self-declaration, ensuring objective classification criteria.

#### 2.2.2 Stress data processing

The raw academic stress data were processed and classified to prepare the input parameters for Data Mining. All stress data corresponding to semester 2024-II were identified by their level. Surveys containing incomplete or inconsistent data were excluded, resulting in 287 valid responses for analysis.

Stress levels were categorized using 20 key variables defined as attributes within the WEKA data mining framework. WEKA provides a robust framework for analyzing and classifying data, allowing the integration of multiple attributes to identify patterns and relationships (Witten et al., 2011).

These 20 essential attributes include variables directly related to stress level (Area of study, Training cycle, Number of courses enrolled, Hours of study, Age, Gender, Marital status, Number of children, Academic performance, Level of worry or nervousness, Level of stressors, Physical reactions, Psychological reactions, Behavioral reactions, Strategy 1, Strategy 2, Strategy 3, Strategy 4, Strategy 5, Strategy 6).

When the attributes in Table 1 are analyzed for stress level, the sample presents an almost perfectly balanced distribution between genders, with 50.2% male and 49.8% female. The majority of students (47%) are between 21 and 25 years old, representing the central stage of university life. In the academic cycle, 41.1% are in specialty training, followed by 30% in specific training and 28.9% in general training. This distribution reflects different moments in the academic trajectory, with a slight predominance of students in more advanced stages of their careers. The area of training shows a clear predominance of Engineering and Architecture with 40.1%, followed by Accounting, Economics, and Administrative Sciences with 31%. Social Sciences (15%) and Health Sciences (13.9%) have a lower representation, which could indicate the profile of the educational institution or the most popular careers. Almost all are single (96.2%), reflecting the typical profile of university students. An interesting fact is that only 9.8% belong to vulnerable populations. Some 93.7% experience moments of nervousness throughout the semester, which reflects that almost all are going through situations that generate considerable anxiety and tension. 39.4% report a moderate level of worry, while 30% indicate that to are quite worried. This suggests that more than 69% of students are experiencing considerable levels of stress that could impact their academic performance and personal wellbeing. In terms of stressors, 60.3% are at a medium level, while 24.4% perceive them at a high level. Reactions are similarly distributed, physical (51.6% medium), psychological (45.6% medium), and behavioral (45.6% medium). This is evidence that students are experiencing considerable impact in terms of stress, affecting aspects such as their health, academic performance, and behavioral conditions.

Table 2 presents the coping strategies used by the students, revealing that the majority employ methods of moderate intensity. Some 38.7% of the students indicate that they “sometimes” defend their preferences or feelings without harming others, while 39.4% elaborate occasional plans to organize their tasks. In addition, 33.8% occasionally seek information about their stressful situations. On the other hand, 35.2% of the students engage “sometimes” in self-praise, which could reflect an attempt at self-confidence, while 27.2% also engage in religious practice or attend church/temple in stressful situations, albeit less frequently. Finally, 33.1% of students verbalize their concerns and seek emotional support from others “sometimes.” These results suggest that students are developing strategies to cope with stress, but also highlight the need for more support and tools to effectively manage these situations.

**Table 2:** Coping strategies.

**Variable**	**Frequency (*n* = 287)**	**Percentage**
**Strategy 1: Ability to defend our preferences or feelings**		
**without harming others**		
Never	28	9.8
Rarely	58	20.2
Sometimes	111	38.7
Almost always	70	24.4
Always	20	7.0
**Strategy 2: Development of a plan for the execution of your**		
**tasks**		
Never	18	6.3
Rarely	78	27.2
Sometimes	113	39.4
Almost always	62	21.6
Always	16	5.6
**Strategy 3: Self-praise**		
Never	46	16.0
Rarely	74	25.8
Sometimes	101	35.2
Almost always	40	13.9
Always	26	9.1
**Strategy 4: Religious practice (prayers or church/temple**		
**attendance)**		
Never	65	22.6
Rarely	90	31.4
Sometimes	78	27.2
Almost always	35	12.2
Always	19	6.6
**Strategy 5: Search for information about the situation**		
Never	38	13.2
Rarely	75	26.1
Sometimes	97	33.8
Almost always	59	20.6
Always	18	6.3
**Strategy 6: Ventilation and confidence (verbalization of the**		
**situation of concern)**		
Never	47	16.4
Rarely	96	33.4
Sometimes	95	33.1
Almost always	27	9.4
Always	22	7.7

**Figure 2:**
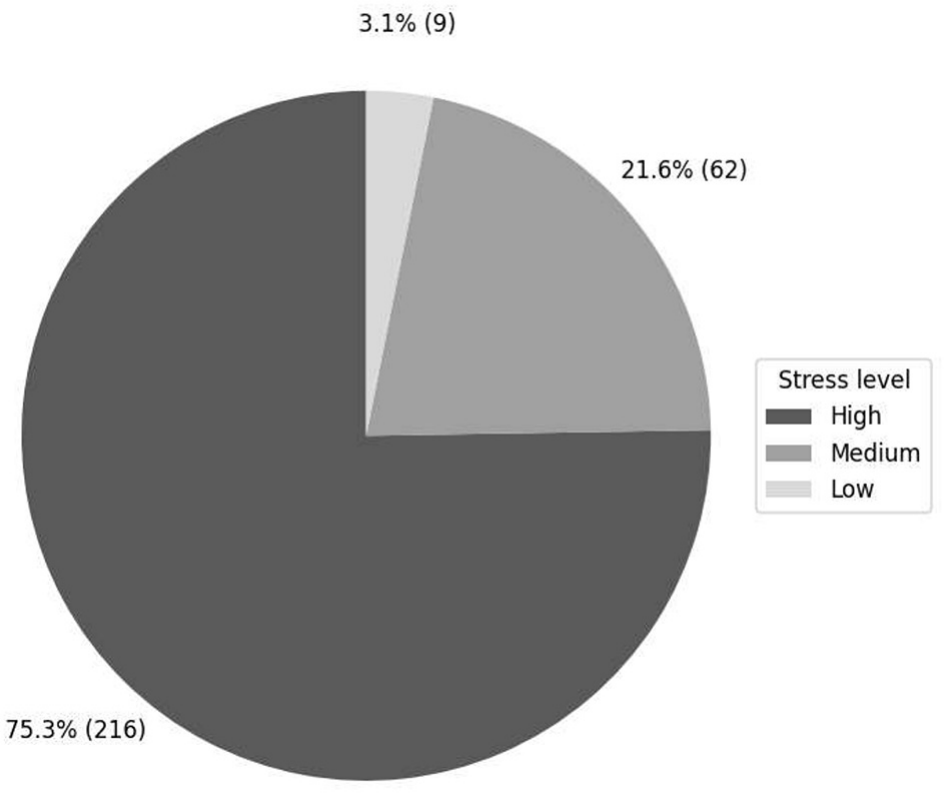
Stress levels.

In Figure 2, the level of stress is particularly alarming: 75.3% of students present a high level of stress, which means that three out of four students are in a condition of high emotional and psychological vulnerability. This percentage indicates a structural problem in the educational system that requires immediate attention.

#### 2.2.3 Use of data mining algorithms

The implementation of data mining was performed in two phases: In the first, algorithms were applied to classify and predict stress level data; the objective was to reveal the ability of data mining algorithms to classify and predict stress levels. In the second stage, association rules were applied to stress level data; the objective was to reveal hidden relationships between variables in the form of rules.

To ensure robustness, a wide range of classification algorithms was selected, including trees, rule-based models, probabilistic approaches, ensembles, and logistic methods. This diversity allows for comparisons across different paradigms, avoiding dependence on the strengths or weaknesses of a single model and aligning with recommendations in recent educational data mining studies (Corrales et al., 2021; Khairy et al., 2024). The data mining classification algorithms employed were:

**J48:** A machine learning algorithm used in data mining to create decision trees. It uses a training dataset to build a tree to make rule-based decisions. Each node of the tree represents a feature of the data and branches according to criteria that maximize the homogeneity of the classes. These trees are used to classify new data instances or predict outcomes based on their characteristics. J48 is a specific implementation of C4.5 in the Weka software (Witten et al., 2016; Saranaval and Gayathri, 2018).

**LMT (Logistic Model Tree):** Provides a very good description of the data. It consists of a decision tree structure with logistic regression functions at the leaves (McMahan et al., 2005). As in ordinary decision trees, a test on one of the attributes is associated with each internal node (Fayaz et al., 2021; Landwehr et al., 2005).

**SimpleLogistic:** A statistical model for binary classification using the probability that an observation belongs to a class. The SimpleLogistic classifier simply implements this model, providing fast results for classification problems (Ali, 2021; Witten et al., 2011).

**RandomForest:** It is an ensemble model based on the construction of multiple decision trees from random subsets of data. Each tree is trained independently, and its predictions are combined to obtain a robust final result (Breiman, 2001).

**REPTree:** It is an efficient implementation of decision trees, which uses classification error reduction to build trees more quickly, making them suitable for large volumes of data (Hall et al., 2009).

**DecisionTable:** Uses a decision table to represent the rules that determine the output as a function of the inputs. It is suitable for problems where the relationship between inputs and outputs is simple and well-defined (Witten et al., 2016).

**Bagging:** Bagging, or Bootstrap Aggregating, is an ensemble method that trains multiple models on random subsets of the data and then averages or votes their predictions to improve model accuracy. This approach is effective in reducing model variance and improving model stability and generalization (Breiman, 1996).

**Bayes Network Classifier (BayesNet):** Bayesian networks are probabilistic models that represent dependency relationships between variables. These models allow inferences and classifications based on probability distributions, being useful in cases where variables are interrelated (Heckerman, 1997).

**NaiveBayes:** A classifier based on Bayes' theorem that assumes that features are independent of each other. Despite this simplification, the algorithm often provides good results in classification tasks (Rennie et al., 2003).

**SMO (Sequential Minimal Optimization):** An algorithm designed to train support vector machines (SVM) efficiently, solving quadratic optimization problems in minimal steps, which makes training much faster and suitable for large data sets (Platt, 1998).

**JRip:** An algorithm that generates classification rules based on the RIPPER algorithm, which divides data into attributes and constructs logical rules for prediction (Cohen, 1995).

**DecisionStump:** It is a simple classifier that uses a single attribute of the data to split it into two groups, creating a decision tree with a single node. Despite its simplicity, it is mainly used as a base classifier in ensemble techniques such as AdaBoost, where several weak models (such as DecisionStump) are combined to improve performance (Freund and Schapire, 1996).

**Locally weighted learning (LWL):** A learning approach that fits prediction models for data near a specific query point. This approach allows the model to better adapt to local variations in the data, making it especially useful when relationships in the data are not homogeneous across the feature space. LWL adjusts the model based on the closeness of the data to the query point, which improves the accuracy of local predictions (Atkeson et al., 1997).

**PART:** It is a hybrid algorithm that combines rule induction with decision tree construction. This algorithm generates a set of classification rules based on a decision tree, which are then interpreted to make decisions. The advantage of PART is that it produces rules that are easy to understand, which facilitate the interpretation of the model's decisions (Ibarguren et al., 2018).

The classification processes were performed using data mining algorithms on the stress level database for the 2024-II semester. The classification has two main objectives. The first is to determine the algorithm that best represents all the data. The other is to evaluate the ability of the data mining algorithms to predict the desired parameter.

In association rule mining, the Apriori and predictive Apriori algorithms were applied. The attribute distributions of both algorithms were analyzed. In this way, deeper insights into the level of academic stress were discovered. The Apriori algorithm finds the most frequent attributes in the dataset and generates association rules with these attributes (Chen and Yin, 2022). When generating the rules with the algorithm, the confidence criterion determined in the implementation is taken into account.

#### 2.2.4 Evaluation of results

For classification, cross-validation was used as a test method in the modeling studies of this phase. In addition to these test methods, Kappa, mean absolute error (MAE), and root mean square error (RMSE). The accuracy of the algorithms is determined by the percentage of correctly classified data in the dataset. Considering only the performance, some modifications were made to some parameters to obtain better results.

For the association rule, three criteria were used to identify the relationship: support, trust, and elevation.

***Support:*** It is the percentage of transactions in the database that contain both the set of elements A and B. The degree of support A⇒ B in the rule A⇒ B is the probability that a given set of elements contains A and B, which is expressed by the probability value P(A∪B) (Prajapati et al., 2017). A high degree of support indicates that the mining results are consistent and that the provided rules are effective association rules. On the other hand, a low degree of support indicates that the data mining results appear only occasionally and the provided rules have little value for research. Equation 1 represents the definition of association rule support between A and B (Valdivia et al., 2020).


$$\begin{array}{l} Support\;A\;\Rightarrow B=P\;\left(A\cap B\right)=\;\frac{\left|A\cup B\right|}{\left|D\right|} \end{array}$$ (1)

***Confidence:*** The percentage of database transactions D with item set A that also contains item set B (Han et al., 2012). Confidence is calculated using conditional probability and is expressed relative to the support of the item set (Agrawal et al., 1993) and is represented by Equation 2:


$$\begin{array}{l} Confidence\;A\;\Rightarrow B=\;\frac{Support\;\left(A\;\Rightarrow B\right)}{Support\;\left(A\right)}=\frac{P\;\left(A\cap B\right)}{P\left(A\right)} \end{array}$$ (2)

In Equation 2, support (A ⇒ B) is the number of transactions containing the set of items A and B, and support (A) is the number of transactions containing the set of items A (Prajapati et al., 2017).

***Lift:*** It is used to measure the frequency of A and B together if both sets of elements are statistically independent of each other (Brijs et al., 2003). The calculation is shown in Equation 3:


$$\begin{array}{l} Lift\;A\Rightarrow \;B=\;\frac{Confidence\;\left(A\;\Rightarrow B\right)}{Support\;\left(A\right)}=\frac{P\;\left(A\cap B\right)}{P\left(A\right)P\left(B\right)} \end{array}$$ (3)

The elevation of the rule A ⇒ B shows how much the probability of B will increase if A occurs (Lee et al., 2019). There are three cases:

- When the elevation (A ⇒ B) > 1, then there is a positive interdependence between the antecedent and the consequent; therefore, the rule is considered valuable.
- When the elevation (A ⇒ B) < 1, then there is a negative interdependence between the antecedent and the consequent.
- When (A ⇒ B) = 1, then A and B are independent and there is no correlation between them.

Therefore, the higher the elevation measure, the higher the interest in the generated rules. Thus, with the help of this, it will rank the rules that meet the minimum thresholds of support and confidence (Huaman et al., 2024).

## 3 Results

**Figure 3:**
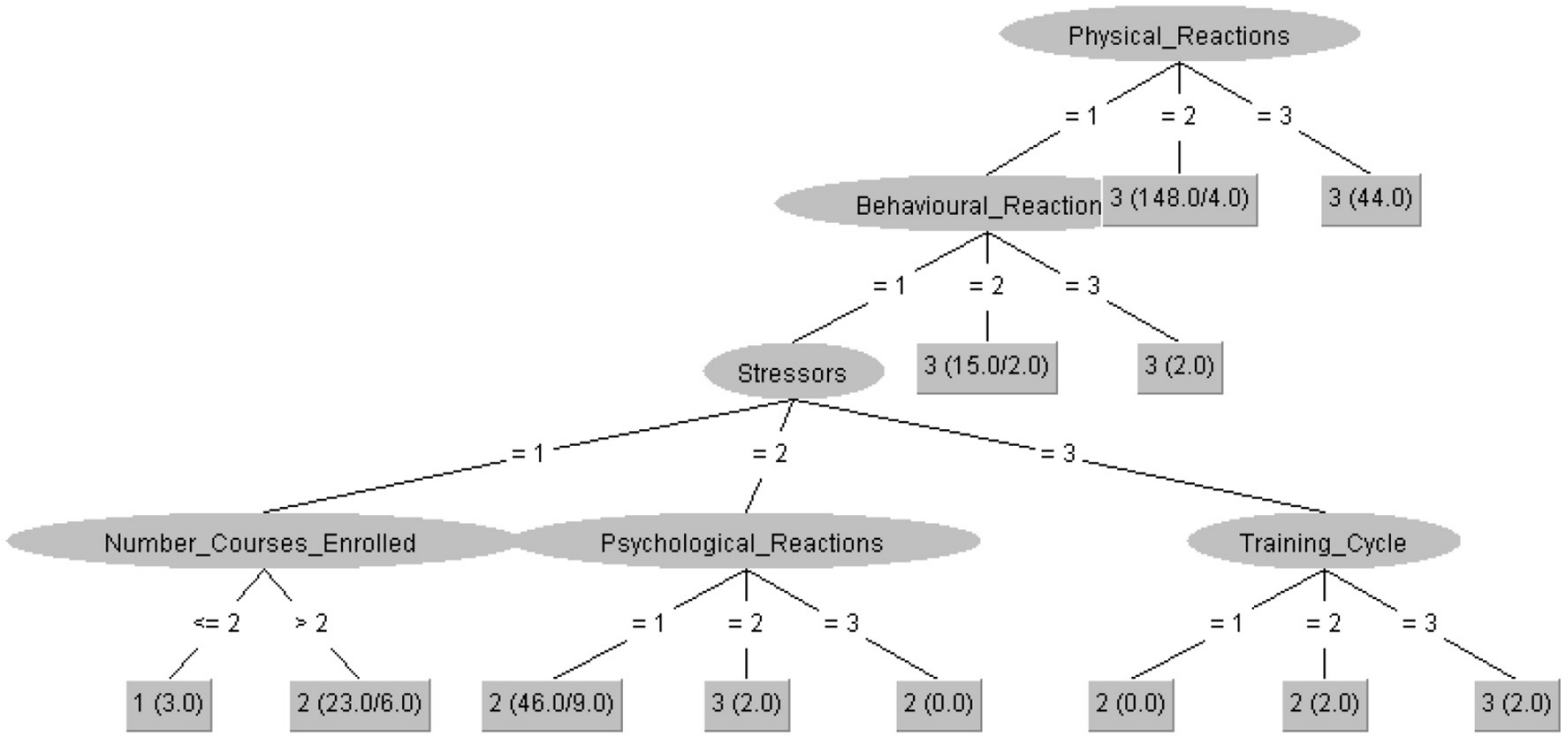
Decision tree J48, for stress level. The terminal leaves display the numbers 1, 2, and 3, which correspond to the stress levels classified according to the SISCO Academic Stress Inventory: 1 = Low stress, 2 = Medium stress, and 3 = High stress. Each branch leads to a final prediction of the students' stress level based on their academic and personal characteristics.

Algorithm J48 with a correct classification percentage of 89.55% shows that students with high stress levels tended to exhibit medium levels of psychological reactions, medium stressors, low behavioral reactions, and low physical reactions. Likewise, students present a high level of stress when they are in the specialty cycle, with a high level of stressors, a low level of behavioral reactions, and a low level of physical reactions (see Figure 3).

In the context of stress level analysis, LMT classifies students into three classes, using an equation for each class based on variables such as nervousness, stressors, and physical, psychological, and behavioral reactions. The results for LMT also showed a correct classification rate of 89.55%.


$$\begin{array}{l} Class\;1:-3.64\;+\;\left[Level\text{\_}Nervousness=1\right]*1.34\\ \;+\left[Stressors=1\right]*2.54\;+\;\left[Psychological\text{\_}Reactions\;=1\right]\\ \;*0.92\\ Class\;2:-1.58\;+\;\left[Level\text{\_}Nervousness=5\right]*-1.25\\ \;+\left[Physical\text{\_}Reactions=1\right]*1.77\\ \;+\left[Behavioural\text{\_}Reactions=1\right]*1.25\;+\left[Strategy\text{\_}2=4\right]\\ \;*-1.01\;+\left[Strategy\text{\_}6=4\right]*-0.78\\ Class\;3:4.58\;+\;\left[Stressors=1\right]*-0.98\;\left[Physical\text{\_}Reactions=1\right]\\ \;*-2.05\;+\;\left[Psychological\text{\_}Reactions\;=1\right]*-1.61\\ \;+\left[Behavioural\text{\_}Reactions=1\right]*-1.18 \end{array}$$

In the stress level analysis, the SimpleLogistic classifier algorithm predicts students' classes using logistic regression, based on the variables in the model, with 89.55% in terms of correct classification.


$$\begin{array}{l} Class\;1:-3.64\;+\;\left[Level\text{\_}Nervousness=1\right]*1.34\\ \;+\;\left[Stressors=1\right]*2.54\;+\;[Psychological\text{\_}Reactions\\ \;=1]*0.92\\ Class\;2:-1.58\;+\;\left[Level\text{\_}Nervousness=5\right]*-1.25\\ \;+\left[Physical\text{\_}Reactions=1\right]*1.77\\ \;+{\left[Behavioural\text{\_}Reactions=1\right]}^{*}1.25\;+\left[Strategy\text{\_}2=4\right]\\ \;*-1.01\;+{\left[Strategy\text{\_}6=4\right]}^{*}-0.78\\ Class\;3:4.58\;+\;\left[Stresors=1\right]*-0.98\;+\;[Physical\text{\_}Reactions\\ \;=1]*-2.05\;+\left[Psychological\text{\_}Reactions\;=1\right]*-1.61\\ \;+\left[Behavioural\text{\_}Reactions=1\right]*-1.18 \end{array}$$

Although simple, JRip proved effective in terms of both accuracy and interpretability. The stress level analysis correctly classified a significant percentage of students, with a correct classification score of 88.85%.

In the student stress analysis, RandomForest showed a correct rating of 88.85%.

**Figure 4:**
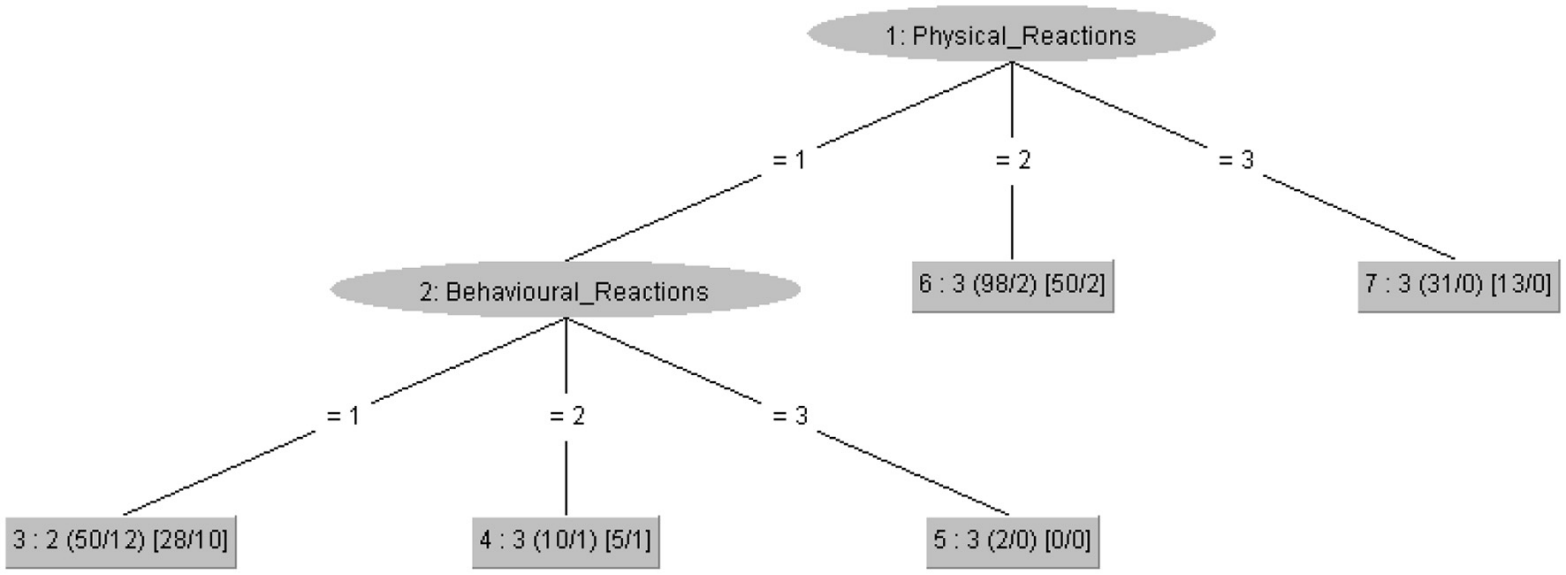
REPTree decision tree for stress level. The numerical values at the leaves indicate the predicted stress level: 1 = Low stress, 2 = Medium stress, and 3 = High stress. The interpretation of each branch shows how combinations of physical, psychological, and behavioral reactions determine the final classification of students' stress levels.

In the stress analysis, REPTree obtained a percentage of correct classification of 88.50%, showing that students with high stress levels tended to exhibit medium or high behavioral reactions, and a low level in their physical reactions (see Figure 4).

This model is suitable for problems where the relationships between attributes and classes are easily interpreted. DecisionTable obtained a correct classification rate of 88.15% in the stress analysis.

The Bagging or Bootstrap Aggregating method reduces variance and improves the stability of the results. The stress analysis showed a performance similar to the correct classification of 88.50%.

The Bayes Network Classifier (BayesNet) approach is useful for modeling and classifying data with complex relationships between variables. In the stress analysis, it showed a correct classification rate of 88.50%.

Although this assumption is rarely realistic, NaiveBayes is still effective for classification tasks, especially on high-dimensional problems. In the stress analysis, a correct classification rate of 87.80% was obtained.

The SMO algorithm, for the analysis of students' academic stress levels, showed a correct classification of 86.76%.

In the stress level analysis, DecisionStump showed a correct classification rate of 85.71%, reflecting its ability to contribute to an effective assembly model.

In the stress analysis, the LWL showed a correct classification rate of 85.71%, adapting well to variations in the data.

Despite being relatively simple, the PART algorithm has proven to be effective in data classification. In the stress analysis, it obtained a correct classification rate of 84.32%.

Table 3 shows that all applied algorithms achieved over 84% correct classification, confirming robust capacity to identify academic stress patterns in students under WEKA's default hyperparameters. The top-performing models were J48, LMT, and SimpleLogistic, each with 89.55% correct classification and a Kappa index of 0.74, reflecting substantial agreement beyond chance. Similarly, JRip (88.85%), RandomForest (88.55%), and REPTree (88.50%) exceeded 88%, while DecisionTable, Bagging, and BayesNet remained close to 88%. In the lower but still acceptable range were NaiveBayes, SMO, DecisionStump, LWL, and PART. Error metrics (MAE and RMSE) were generally low, though slightly higher for SMO.

**Table 3:** Algorithms applied and results obtained.

**Algorithm**	**Correct classified (%)**	**Kappa**	**MAE**	**RMSE**	**Classification correct**	**Classification incorrect**
J48	89.55	0.74	0.10	0.24	257	30
LMT	89.55	0.74	0.10	0.23	257	30
SimpleLogistic	89.55	0.74	0.10	0.23	257	30
JRip	88.85	0.72	0.10	0.25	255	32
RandomForest	88.55	0.70	0.13	0.24	255	32
REPTree	88.50	0.70	0.11	0.25	254	33
DecisionTable	88.15	0.71	0.15	0.25	253	34
Bagging	88.50	0.71	0.13	0.25	254	33
BayesNet	88.50	0.73	0.09	0.25	254	33
NaiveBayes	87.80	0.71	0.09	0.25	252	35
SMO	86.76	0.66	0.25	0.32	249	38
DecisionStump	85.71	0.66	0.14	0.26	246	41
LWL	85.71	0.66	0.13	0.26	246	41
PART	84.32	0.60	0.11	0.28	242	45

All algorithms were executed with the default hyperparameter settings in WEKA, which ensured methodological transparency, reproducibility of results, and comparability with previous studies. The purpose was to evaluate the classifiers under standardized and replicable conditions rather than optimizing the performance of individual models.

Beyond numerical accuracy, the models reveal that academic stress results from the interaction of multiple factors. The decision tree J48 highlights the importance of the specialty cycle and level of stressors; LMT and SimpleLogistic emphasize nervousness and psychological reactions as decisive predictors; and other models reinforce the role of behavioral and physical responses. Taken together, the findings underscore that students in advanced academic stages, facing cumulative stressors and lacking strong adaptive responses, are particularly vulnerable to experiencing high levels of stress.

Table 4 presents the precision, recall, and other performance metrics by class for the evaluated algorithms. The results show strong performance in identifying the high-stress class (class 3), with recall and F-measure values generally above 0.90, indicating that the models accurately capture the patterns associated with this category. The medium-stress class (class 2) achieves intermediate levels of performance, with recall values ranging from 0.74 to 0.87 depending on the algorithm, reflecting acceptable detection rates though with a higher risk of misclassification. The lowest results are observed in the low-stress class (class 1), where recall and F-measure remain between 0.00 and 0.22, highlighting the challenge of accurately detecting cases in this group. The metrics reflect consistent algorithmic behavior, with greater reliability in the categories that show clearer and more distinguishable patterns.

**Table 4:** Class-level precision, recall, and performance metrics of classification algorithms.

**Algorithm**	**TP Rate**	**FP Rate**	**Precision**	**Recall**	**F-Measure**	**MCC**	**ROC Area**	**PRC Area**	**Class**
**J48**	0.11	0.00	0.50	0.11	0.18	0.23	0.90	0.25	1
	0.87	0.10	0.71	0.87	0.78	0.72	0.89	0.65	2
	0.94	0.10	0.97	0.94	0.95	0.81	0.94	0.97	3
Weighted Avg.	0.90	0.10	0.90	0.90	0.89	0.77	0.93	0.88	
**LMT**	0.00	0.01	0.00	0.00	0.00	−0.02	0.92	0.21	1
	0.86	0.09	0.72	0.86	0.78	0.72	0.94	0.67	2
	0.94	0.07	0.98	0.94	0.96	0.85	0.97	0.99	3
Weighted Avg.	0.90	0.07	0.89	0.90	0.89	0.79	0.96	0.90	
**SimpleLogistic**	0.00	0.01	0.00	0.00	0.00	−0.02	0.92	0.21	1
	0.86	0.09	0.72	0.86	0.78	0.72	0.94	0.67	2
	0.94	0.07	0.98	0.94	0.96	0.85	0.97	0.99	3
Weighted Avg.	0.90	0.07	0.89	0.90	0.89	0.79	0.96	0.90	
**JRip**	0.22	0.02	0.25	0.22	0.24	0.21	0.87	0.19	1
	0.89	0.09	0.73	0.89	0.80	0.75	0.90	0.69	2
	0.92	0.09	0.97	0.92	0.94	0.79	0.91	0.96	3
Weighted Avg.	0.89	0.08	0.90	0.89	0.89	0.76	0.91	0.88	
**RandomForest**	0.11	0.01	0.33	0.11	0.17	0.18	0.92	0.26	1
	0.73	0.07	0.75	0.73	0.74	0.67	0.94	0.70	2
	0.97	0.21	0.93	0.97	0.95	0.79	0.97	0.99	3
Weighted Avg.	0.89	0.17	0.88	0.89	0.88	0.74	0.96	0.91	
**REPTree**	0.00	0.00		0.00			0.87	0.17	1
	0.86	0.10	0.70	0.86	0.77	0.70	0.90	0.67	2
	0.93	0.14	0.95	0.93	0.94	0.77	0.93	0.97	3
Weighted Avg.	0.89	0.13		0.89			0.92	0.88	
**DecisionTable**	0.00	0.02	0.00	0.00	0.00	−0.02	0.82	0.08	1
	0.87	0.10	0.71	0.87	0.78	0.72	0.92	0.67	2
	0.92	0.10	0.97	0.92	0.94	0.79	0.95	0.98	3
Weighted Avg.	0.88	0.10	0.88	0.88	0.88	0.75	0.94	0.89	
**Bagging**	0.11	0.01	0.33	0.11	0.17	0.18	0.93	0.30	1
	0.82	0.10	0.70	0.82	0.76	0.69	0.92	0.66	2
	0.94	0.13	0.96	0.94	0.95	0.79	0.95	0.98	3
Weighted Avg.	0.89	0.12	0.88	0.89	0.88	0.75	0.94	0.89	
**BayesNet**	0.33	0.03	0.27	0.33	0.30	0.28	0.91	0.30	1
	0.87	0.10	0.70	0.87	0.78	0.71	0.95	0.75	2
	0.91	0.03	0.99	0.91	0.95	0.83	0.98	0.99	3
Weighted Avg.	0.89	0.04	0.91	0.89	0.89	0.79	0.97	0.92	
**NaiveBayes**	0.22	0.04	0.17	0.22	0.19	0.16	0.90	0.25	1
	0.87	0.10	0.70	0.87	0.78	0.71	0.95	0.73	2
	0.91	0.03	0.99	0.91	0.95	0.82	0.98	0.99	3
Weighted Avg.	0.88	0.04	0.90	0.88	0.89	0.78	0.97	0.91	
**SMO**	0.11	0.02	0.17	0.11	0.13	0.11	0.91	0.29	1
	0.74	0.10	0.68	0.74	0.71	0.62	0.82	0.56	2
	0.94	0.16	0.95	0.94	0.94	0.77	0.90	0.94	3
Weighted Avg.	0.87	0.14	0.87	0.87	0.87	0.72	0.88	0.84	
**DecisionStump**	0.00	0.00		0.00			0.82	0.09	1
	0.94	0.16	0.61	0.94	0.74	0.67	0.86	0.55	2
	0.87	0.06	0.98	0.87	0.92	0.75	0.88	0.94	3
Weighted Avg.	0.86	0.08		0.86			0.87	0.83	
**LWL**	0.00	0.00		0.00			0.95	0.29	1
	0.92	0.16	0.61	0.92	0.74	0.67	0.92	0.65	2
	0.88	0.07	0.97	0.88	0.92	0.74	0.95	0.99	3
Weighted Avg.	0.86	0.09		0.86			0.95	0.89	
**PART**	0.33	0.04	0.23	0.33	0.27	0.25	0.85	0.21	1
	0.66	0.09	0.66	0.66	0.66	0.57	0.90	0.65	2
	0.92	0.20	0.93	0.92	0.93	0.71	0.93	0.97	3
Weighted Avg.	0.84	0.17	0.85	0.84	0.85	0.66	0.92	0.88	

Class 1 corresponds to the low-stress level, class 2 to the medium-stress level, and class 3 to the high-stress level.

Table 5 shows the confusion matrices for the evaluated algorithms. The high-stress class (class 3) accounts for most of the correct classifications, confirming consistency with the high recall values obtained. In the medium-stress class (class 2), a significant number of cases were misclassified as high stress, indicating that students at the intermediate level tend to be classified as higher risk. The low-stress class (class 1) is the most affected, as most of its cases were assigned to the higher categories, with few correct predictions, showing the difficulty of the models in distinguishing this group. The confusion matrices demonstrate solid performance in the majority category but also reveal limitations in differentiating lower stress levels.

**Table 5:** Confusion matrix of classification algorithms applied to academic stress level.

**Algorithm**	**a**	**b**	**c**	**classified as**
J48	1	8	0	a = 1
	1	54	7	b = 2
	0	14	202	c = 3
LMT	0	9	0	a = 1
	4	53	5	b = 2
	0	12	204	c = 3
SimpleLogistic	0	9	0	a = 1
	4	53	5	b = 2
	0	12	204	c = 3
JRip	2	7	0	a = 1
	1	55	6	b = 2
	5	13	198	c = 3
RandomForest	1	8	0	a = 1
	2	45	15	b = 2
	0	7	209	c = 3
REPTree	0	8	1	a = 1
	0	53	9	b = 2
	0	15	201	c = 3
DecisionTable	0	9	0	a = 1
	1	54	7	b = 2
	4	13	199	c = 3
Bagging	1	8	0	a = 1
	2	51	9	b = 2
	0	14	202	c = 3
BayesNet	3	6	0	a = 1
	6	54	2	b = 2
	2	17	197	c = 3
NaiveBayes	2	7	0	a = 1
	6	54	2	b = 2
	4	16	196	c = 3
SMO	1	8	0	a = 1
	5	46	11	b = 2
	0	14	202	c = 3
DecisionStump	0	9	0	a = 1
	0	58	4	b = 2
	0	28	188	c = 3
LWL	0	9	0	a = 1
	0	57	5	b = 2
	0	27	189	c = 3
PART	3	6	0	a = 1
	7	41	14	b = 2
	3	15	198	c = 3

In the confusion matrix: a = 1 corresponds to the low-stress level, b = 2 to the medium-stress level, and c = 3 to the high-stress level.

Table 6 presents the association rules generated using the Apriori algorithm, applied to explore the relationships between variables such as number of children, stress level, stressors, and marital status in college students. The table's columns show key metrics to assess the strength of the associations: confidence, lift, leverage, and convergence. It is observed that the rules with the highest level of confidence, especially those relating to marital status and number of children, also present a high lift, indicating a significant relationship between the variables. These results allow us to identify relevant patterns of personal conditions associated with the level of stress in the population studied.

**Table 6:** Association rules generated using the Apriori algorithm.

**No**.	**Association rules**	**Support**	**Confidence**	**Lift**	**Leverage**	**Convergence**
1	Number_Children = 0 Stressors = 2 = => Marital_status = 1	161	1	1.04	0.02	6.17
2	Number_Children = 0 = => Marital_status = 1	266	1	1.04	0.03	5.12
3	Number_Children = 0 Stress_Level = 3 = => Marital_status = 1	201	1	1.03	0.02	3.87
4	Stressors = 2 = => Marital_status = 1	168	0.97	1.01	0.01	1.11
5	Marital_status = 1 Stress_Level = 3 = => Number_Children = 0	201	0.97	1.04	0.03	2.06
6	Marital_status = 1 276 = => Number_Children = 0	266	0.96	1.04	0.03	1.75
7	Stress_Level = 3 = => Marital_status = 1	207	0.96	1	−0	0.83
8	Marital_status = 1 Stressors = 2 = => Number_Children = 0	161	0.96	1.03	0.02	1.46
9	Stress_Level = 3 = => Number_Children = 0	202	0.94	1.01	0	1
10	Stressors = 2 = => Number_Children = 0	161	0.93	1	0	0.93

The association rules were generated using the Apriori algorithm in WEKA with default parameters.

The results of the Apriori association analysis reveal that, considering stress level as the dependent variable, there is a strong correlation between a high level of stress (Stress_Level = 3) and certain demographic characteristics of the participants. Specifically, 94% of high-stress people have no children (Number_Children = 0), and 96% have a specific marital status (Marital_status = 1), “single.” This association suggests that single people without children are more likely to experience elevated levels of stress, which may reflect factors such as work or academic pressures that significantly affect students with these demographic characteristics.

This finding shows that, in the analyzed population, the absence of direct family support is linked to greater vulnerability to academic demands. The recurrence of these conditions across different rules confirms that academic stress does not depend solely on academic factors but is reinforced by personal characteristics. In other words, young, single, and childless students lack support networks that could buffer the emotional impact of university workload, making them particularly prone to experiencing high levels of stress. In this sense, these results provide evidence for designing preventive interventions targeted at these profiles, considering both the academic context and the personal factors that increase vulnerability.

## 4 Discussion

Analysis of data collected from 287 university students showed that 75.3% experienced high stress levels, 21.6% moderate stress, and only 3.1% low stress. These results indicate that three out of four students were highly vulnerable emotionally and psychologically within the university environment, which is consistent with previous studies that have identified higher education as a particularly critical stage for mental health (Bewick et al., 2010; Bonneville-Roussy et al., 2017). Similarly, research such as Cuttilan et al. (2016) reports worrying levels of stress, although the percentage observed in our sample is even higher, evidencing the particular severity in our academic context.

In terms of predictive performance, the applied data mining algorithms (J48, LMT, and SimpleLogistic) achieved classification accuracies above 89%, demonstrating a strong ability to identify stress-level patterns. This robust performance reinforces the reliability of the findings and aligns with previous research supporting the application of data mining techniques in student mental health analysis (Corrales et al., 2021; de la Fuente et al., 2020; Khairy et al., 2024). This level of accuracy is comparable to the results of Corrales et al. (2021), who, using logistic regression and classification trees, achieved accuracies of 78%, highlighting the robustness of data mining tools in this type of educational research.

The analysis of coping strategies revealed that although students employ certain mechanisms, these are inconsistent and often ineffective. For example, only 39.4% occasionally made plans to manage stress, while 33.8% sometimes sought information about their stressful situations. This trend suggests an intermittent use of coping strategies, which is consistent with Nguyen-Thi et al. (2024) and Frydenberg (2014), who warn that without consistent adaptive strategies, students remain vulnerable to the negative effects of academic stress. Additionally, practices such as emotional venting or the use of religious support are even less frequent, limiting avenues for emotional release in the face of university pressure.

The association rules generated using the Apriori algorithm show particularly revealing patterns, single (Marital_Status = 1) and childless (Number_Children = 0) students are highly associated with an elevated level of stress (Stress_Level = 3). High confidence values (≥96%) and lift scores above 1 further reinforce the significance of these relationships, indicating that such personal characteristics substantially increase the likelihood of experiencing stress. These findings are consistent with previous studies emphasizing the role of social support in mitigating academic stress (Gogichadze et al., 2023; Maity et al., 2022; Ragab et al., 2021). They agree with what was reported in the study by Corrales et al. (2021), where personal and contextual conditions (such as lack of social support and high academic demands) were found to be key determinants of students' stress levels. In addition, the lack of family responsibilities could imply a greater dedication to academic life, which, although beneficial in terms of concentration, can translate into more intense pressure, economic worries, and anxiety about the future.

The association rules, characterized by high confidence and lift values, suggest that the absence of personal support networks defines a particularly vulnerable student profile to the demands of the university environment. These characteristics, rather than functioning as simple background variables, indicate greater vulnerability to institutional pressures in the absence of social and emotional support. Close relationships and family responsibilities may act as protective factors that buffer stress (Gogichadze et al., 2023; Maity et al., 2022). This interpretation aligns with the findings of Li and Hasson (2020) and Hitches et al. (2022), who argue that resilience to stress depends not only on individual competencies but also on affective bonds and contextual resources. Therefore, academic stress should not be seen merely as a consequence of external or familial factors, but as a systemic experience within higher education, one that universities must recognize and address as an institutional responsibility.

The elevated stress levels experienced by single, childless students in college settings can be attributed to a variety of factors. These include academic pressures, financial concerns, and personal lifestyle choices, thereby contributing to a challenging environment for students (Cody et al., 2024). Understanding these factors is crucial to developing effective interventions to support student wellbeing (Chemagosi, 2024). Students often face intense academic demands, including heavy workloads and high-performance standards, leading to significant stress (Slimmen et al., 2022). Pressure to perform well on exams can exacerbate feelings of anxiety and stress (Islam and Rabbi, 2024). Many students experience financial difficulties, which can generate additional pressure and anxiety, affecting their overall mental health (Mofatteh, 2021). On the other hand, the analysis of associated variables reveals that marital status and childlessness not only describe demographic conditions, but also reflect student profiles with a greater focus on their academic career, while simultaneously exposing them to internal pressures without the emotional buffer that close personal relationships might represent (Hitches et al., 2022; Li and Hasson, 2020).

Higher education benefits the individual, as well as the community and society to which it contributes, however, the educational path is not without challenges (Durán Acevedo et al., 2021). Internationally, students report a high level of stress related to their education, which can have adverse effects on their health, quality of life, and academic achievement (Pascoe et al., 2020). However, when students are academically confident, they experience less stress, adapt more successfully to college, and are considered “healthier” and “happier” individuals (Chemers et al., 2001). Success in higher education encompasses not only student achievement but also satisfaction with life, which academic confidence (self-efficacy) and stress can predict, respectively (Krumrei-Mancuso et al., 2013). Therefore, examining the latter factors may provide information on how best to support college students to reach their full potential and which individuals may most need such support (Hitches et al., 2022).

Furthermore, the results show that academic stress does not arise as an isolated factor but as a consequence of multiple interrelated dimensions: academic pressure, course load, frequent nervousness, moderate or high physical and psychological reactions, and insufficient coping strategies. All this reinforces the need for a comprehensive approach to university stress prevention and care, as already proposed by authors such as Slavin et al. (2012) and Castillo-Navarrete et al. (2024).

From a theoretical standpoint, the study reinforces the notion of academic stress as a systemic phenomenon, resulting from the interaction between curricular demands, students' resources, and institutional conditions. In line with current models of stress in higher education (Misra and Castillo, 2004; Moradi et al., 2011), it confirms that task overload, evaluation pressure, and internal competition are key triggers of this issue. Moreover, it demonstrates that data mining algorithms provide robust classification accuracy, thereby contributing methodological evidence that enriches the theoretical framework on student stress prediction.

From a practical perspective, the findings highlight the urgent need for preventive policies in curriculum design, academic workload management, and the establishment of permanent emotional support spaces. Additionally, it is recommended to incorporate risk profile analyses, such as marital status or the absence of support networks, into student wellness programs (Kim and Sax, 2017; Stallman, 2010). Universities should implement early intervention systems based on predictive models, develop workshops in socioemotional skills, and create mentoring spaces that strengthen support networks, offering not only crisis management but also preventive and sustainable strategies for student wellbeing.

The evidence gathered in this study makes it clear that academic stress should not be seen as a mere individual reaction to study load, but rather as the result of a deeply rooted institutional construct, driven by structural demands, competitive dynamics, and the lack of effective support mechanisms. This perspective aligns with the findings of Pascoe et al. (2020) and Powell and Graham (2017), who warn about the detrimental impact that the modern educational system can have on students' mental health. In this context, it becomes essential for universities to take on an active and transformative role in promoting student wellbeing by implementing policies that include early emotional support, stress management workshops, socioemotional skills programs, and a redesign of curricular dynamics to make them more humane, balanced, and responsive to students' real needs. Only through these structural changes can we move toward a more sustainable, empathetic educational experience that places mental health at the core of professional development.

However, the results should be interpreted with caution considering certain limitations of the study. First, the data were collected through self-administered questionnaires, which may introduce biases such as social desirability or recall errors (Licht-Ardila et al., 2021). Although a validated instrument such as the SISCO inventory was used, the subjective perception of stress does not always accurately reflect its actual intensity. In addition, since most variables are self-reported and qualitative in nature, there is a risk that responses reflect individual perceptions rather than objective measures, which may weaken the strength of the inferences. Therefore, the conclusions should be understood as indicative of patterns and associations rather than direct causal relationships. Moreover, while data mining algorithms provide strong predictive capabilities, their internal logic is not always fully interpretable, especially in complex models such as ensembles or those based on sequential optimization (Atzmueller et al., 2024; Cortis and Davis, 2021). The study was also cross-sectional, limiting the ability to capture how stress levels fluctuate throughout an academic program. Research by Bewick et al. (2010), for instance, has shown that stress can vary significantly across academic cycles, suggesting that future studies should incorporate longitudinal approaches for a deeper understanding of these dynamics.

Additionally, it must be acknowledged that class imbalance was a methodological limitation. Although tests were conducted using resampling techniques such as SMOTE, undersampling, and cost-sensitive learning, the results did not improve performance on the minority class (low stress) and, in some cases, even reduced overall accuracy. Likewise, macro-averaged metrics were explored for a fairer evaluation across categories, but these highlighted the difficulty of the model in learning patterns from underrepresented classes. Therefore, per-class metrics and weighted averages were reported as the primary reference, while noting that future work should expand the sample or implement more robust hybrid approaches. Transforming the educational experience into a space that values not only academic performance but also the student's overall wellbeing requires universities to assume an active commitment to the creation of emotionally healthy environments, with permanent psychological support programs, preventive stress management workshops, and the promotion of more collaborative and less competitive settings that strengthen socio-emotional skills and peer support networks, thereby contributing to the development of resilient, balanced, and emotionally healthy professionals.

## 5 Conclusions

The results of this study show a worrisome picture, revealing that three out of four students experience high stress levels, affecting both their wellbeing and academic performance. This phenomenon is not isolated, as the majority of respondents also report medium or high levels of physical, psychological, and behavioral reactions to stressful situations. Despite employing some coping strategies, such as occasionally making plans or seeking information, these actions do not seem to be systematic or effective enough to mitigate the impact of stress. In addition, the association rules analysis revealed that single and childless students are the most vulnerable, suggesting that the lack of personal support networks could aggravate the experience of stress in academic life.

Regarding the performance of the data mining algorithms applied, a high accuracy rate was achieved in the prediction of stress levels, exceeding 89% in some cases. Beyond this performance, the main contribution is that these techniques help identify key determinants, such as the academic cycle, stress levels, and emotional reactions, offering a practical framework for prioritizing student support. In particular, models based on decision trees and logistic regression allowed us to observe that factors such as academic cycle, level of stressors, and emotional reactions are major determinants in the classification of stress levels. Taken together, the methodological evidence supports the use of analytics not only to classify risk but also to inform targeted, data-driven decisions in higher education.

Based on these findings, universities must design student welfare policies that address not only critical cases but also preventive measures. Implement continuous emotional support programs, socioemotional skills workshops, and personalized counseling in time management and stress management. Structural adjustments are also recommended, for example, reviewing teaching loads and assessment pressure, to foster more balanced learning environments. Likewise, support networks among students should be strengthened, promoting meeting and mentoring spaces, where students can share experiences, concerns, and coping strategies in a safe and accompanied way. In this approach, mental health should operate as a transversal axis that guides policies, resource allocation, and evaluation of metrics.

This study opens a fertile space for future research on academic stress and its management in higher education. Although data mining algorithms demonstrated great accuracy in classifying stress levels, future work should adopt longitudinal designs to track changes across academic cycles and transition periods. Cross-institutional and cross-regional studies are needed to test generalizability and contextual effects. It is also crucial to examine additional variable external social support, digital habits and resilience, socioeconomic constraints, and prior mental health, within explanatory models. Finally, intervention-based research should link predictive models to early-warning protocols, evaluate effectiveness through academic and wellbeing outcomes (e.g., retention, GPA, help-seeking), and assess ethical, fairness, and interpretability dimensions of algorithmic tools.

## Data Availability

The original contributions presented in the study are included in the article/supplementary material, further inquiries can be directed to the corresponding author.
